# How Effective Are Citizen Scientists at Contributing to Government Tree Health Public Engagement and Surveillance Needs—An Analysis of the UK Open Air Laboratories (OPAL) Survey Model

**DOI:** 10.3390/insects11090550

**Published:** 2020-08-19

**Authors:** David D. Slawson, Andy J. Moffat

**Affiliations:** 1 Centre for Environmental Policy, Imperial College London, London SW7 1NE, UK; 2 Forest Research, Alice Holt, Farnham GU10 4LH, UK; andy.moffat@forestresearch.gov.uk

**Keywords:** citizen science, tree health, pests and diseases, survey, surveillance, government policy, engagement

## Abstract

**Simple Summary:**

The impact on tree health from insect pests and microbial diseases that have moved across country borders has been increasing in recent years. This poses a significant challenge to government authorities, and a number of countries have been examining how effective volunteers from the general public can be in supporting tree health surveillance. Our paper describes a project led by the Open Air Laboratories (OPAL) in the United Kingdom which tested the extent to which the public were motivated to participate in tree health surveillance. It also examined whether ‘citizen scientists’ could provide information which would be of genuine use to officials and scientists responsible for national tree health. The results suggest that there was considerable engagement from the public, who completed over 2800 surveys covering more than 4500 trees. Nevertheless, despite designing the OPAL survey specifically for untrained individuals, the results were only partially of value to tree health specialists. The paper discusses the results and concludes that involving citizens with some existing expert knowledge is probably the most effective way to generate more reliable data. Lay citizens can contribute effectively at critical times when additional surveillance capacity is needed, provided that suitable guidance and support are given.

**Abstract:**

The incidence of tree disease has been increasing in the UK in recent years as a result of a range of alien tree pests and pathogens new to the country. In the early 2010s government staff resources to monitor, identify and eradicate these pathogens were limited, so we tested the efficacy of “citizen scientists” to support these needs. The Open Air Laboratories (OPAL) is a successful citizen science programme launched in 2007, which at that time of launch involved over 650 thousand people in a range of environmental surveys. In 2012–2013, the Food and Environment Research Agency (Fera) and Forest Research staff worked with OPAL and its partners to launch a citizen science tree health survey in Great Britain and this was extended to cover Northern Ireland until it closed in 2019. Over 2800 surveys were completed including records on more than 4500 trees, the majority from urban areas. This paper discusses the results of the survey and their value for the assessment of tree health. It also considers the implications of engagement with the general public for the future of tree health surveillance. Recommendations are made for further development of the OPAL “model” and more generally for the role of citizen science in this important area.

## 1. Introduction

In recent decades, a major threat to tree health in the United Kingdom has been an increase in the number of incursions of new insect pests and pathogens of trees into the country, partly a result of free trade policies [1,2] (Supplementary Figure S1). Like the previous outbreak of Dutch elm diseases in the 1970s, which removed elm from almost all parts of the British landscape [1], the rapid spread of Chalara ash dieback disease since 2012 reminds us, yet again, that our tree stocks can be severely threatened if pests and pathogens arrive in the UK and are detected too late—in 2004, ash was the fifth most common tree species recorded in English towns and cities [3] and in 2012 it was the third most common broadleaved species in Great Britain, covering 157,000 hectares [4].

Foresters and arboriculturists have always been alert to visible signs of tree/forest ill-health, and the effects of pests and diseases (P&Ds). Formal, government-run, tree health surveys included annual surveys of tree health and forest condition, which ran in Great Britain from 1987 to 2006 [5], based on a detailed protocol for assessment of tree condition [6]. In addition, purposeful surveys for specific pests and pathogens listed in national plant health legislation have taken place in woodlands and forests, and on trees elsewhere, as necessary. In parallel, periodic health surveys of urban and rural non-woodland amenity trees were organised from the 1990s [7] until 2005 [8,9]. In contrast to the forest condition surveys, the surveying of amenity trees was undertaken by volunteer observers. Two further formal surveys of urban tree condition (including health) were sponsored by other government departments under the “Trees in Towns” project [3,10].

In recognition of the threats that plant pests and pathogens pose to agriculture, forestry and the environment, an international treaty exists, the International Plant Protection Convention (IPPC), to protect cultivated and wild plants by preventing the introduction and spread of (plant) pests [11]. The IPPC defines a quarantine pest as a pest (including pathogens) of “potential economic importance to the area endangered thereby and not yet present there, or present but not widely distributed and being officially controlled” and places legal responsibilities on countries to conduct surveillance for quarantine pests to detect their occurrence.

Major surveillance programmes have been traditionally conducted by government agencies, such as the Forestry Commission (FC) and Defra’s Plant Health and Seeds Inspectorate (now part of the Animal and Plant Health Agency but formerly part of the Food and Environment Research Agency (Fera). International and national legislation requires officials from Defra and other agencies to check imports of plants and timber coming into their countries and to conduct surveys in their territories in order to check for the presence of pests and pathogens. Rapid identification facilitates the real prospect of eradication. Conversely, if new pests or pathogens arrive undetected and subsequently spread, then eradication may be impossible, and other less desirable management options such as “containment” or “co-existence” may be required. UK governments are also primarily responsible for raising public awareness, traditionally via “Tree Alerts” or “Pest Alerts” in the UK [12].

Nevertheless, the economic recession during the first decade of the new millennium seriously challenged the strength of government officials to undertake all the work necessary for pest and pathogen detection and eradication. It is estimated that the number of front-line official inspectors to inspect all imports coming into Great Britain was c. 100 in 2012 (80 Fera Plant Health and Seeds Inspectorate and 20 Forestry Commission). Around this time, the Government brought forward its “Big Society” concept [13], which included encouraging citizens to take a greater role in their communities and to take more responsibility for “building the Britain they want”—in other words, taking over some of the roles that had previously been undertaken by government.

Against this backdrop, in 2011 Defra and the FC launched the “Action Plan for Tree Health and Plant Biosecurity” (APTHPB) [14]. The APTHPB set out an integrated approach centred around four main themes: “protecting the UK—import controls”; “practical actions”; “public and stakeholder engagement”; and “research opportunities and evidence priorities.” It stated that the actions are “not just for Government to deliver alone” and that “forestry stakeholders and the public all have an interest and responsibility in protecting woodlands.” Given the demise of the annual forest and amenity tree condition surveys mentioned above, together with the limited institutional capacity of governments to provide effective tree health surveillance on their own, it became clear that policies would be needed to enable effective support from these quarters.

The theme of “Public and stakeholder engagement” in the APTHPB sought to develop guidance setting out how society could help. In addition to actions aimed at raising public awareness and strengthening public engagement, specific actions were included to improve the public’s ability to detect and report pests and pathogens and to develop a cadre of professionals and volunteers to act as “eyes and ears” on the ground to support official surveillance. In the same year, Forest Research (the research agency of the Forestry Commission) also initiated a four-year programme during which time a new, coordinated system to facilitate public reporting of pests and pathogens was to be developed. Some testing of citizen science (CS) support for tree health was also taking place under the auspices of the private and charity sectors at this time. Two major UK government-supported CS tree health projects were initiated: Observatree [15]—a network of trained volunteers working under supervision of officials, and OPAL (Open Air Laboratories)—a citizen science project which engaged with the general public in environmental projects.

OPAL is a UK-wide citizen science initiative launched in 2007 and led by Imperial College London. It includes a range of museums, universities and environmental organisations. In 2012, Fera and Forest Research (FR) scientists met with OPAL managers to explore whether OPAL could develop, launch and manage a CS survey focussed on tree health. At that time, OPAL had organised six successful CS surveys covering a range of topics, such as biodiversity and water, soil and air quality, involving over 650 thousand participants [16]. Under a formal partnership agreement, OPAL personnel and support staff worked together with Fera and Forest Research in a Working Group to develop the survey. An advisory board was set up to help steer the project and extend its reach (Supplementary Tables S1 and S2). The OPAL tree health survey was launched in May 2013 and concluded in October 2019. Methodology used in previous OPAL surveys was adapted to meet the needs of the new one, but it also marked a change from previous practice in that there were strong government policy and research elements to the project. It also represented a significant change in government tree health monitoring activities which had previously been conducted only by officials.

A small number of pests and diseases were selected for observation in the survey. These included those that were already known to be present in the UK, where the purpose was to obtain information on their latest distribution. Others that were either absent from the UK or of only limited distribution were also included such that early detection could allow prompt eradication measures to be taken by government officials. Five insect species featured in this “early warning” category, not only because of their potential to cause serious damage to a range of economically and ecologically important tree species but also because they were deemed to be relatively easy for a trained member of the public to identify.

This paper describes the development and execution of the OPAL Tree Health Survey as an example of a citizen science project that involved a large number of participants. Using a summary of the most important results, we analysed whether members of the general public can play an effective role in national tree health surveillance. The paper discusses how lessons learnt from the OPAL survey could help to further develop surveillance systems utilising citizen science support.

## 2. Materials and Methods

The OPAL approach involved production of surveys designed to be self-explanatory and simple to complete so that people of all ages and abilities could participate. They had the dual aims of encouraging more people to explore their local natural environment and at the same time producing data that could be used for research [16]. The main focus for Forest Research and Fera scientists was whether, through a survey using the OPAL methodology, the public could and would contribute to the surveillance of tree pests and diseases (P&Ds), in support of Government policy responsibilities carried out by these organisations.

Early discussions centred on whether a public-facing survey should focus only on new invasive P&Ds, given the importance of early detection. However, it was considered that such a focus would be likely to fail for several reasons. Firstly, encouraging the public to detect P&D that government biosecurity policies were working to prevent in the UK would likely lead to apathy and disappointment when few or no observations could be made. Secondly, focus on tree ill-health would be counterproductive to one of OPAL’s overarching objectives, that of encouraging public appreciation of nature and the outdoors. Thirdly, expecting public participation solely through observation of presence/absence of a pest or disease would be insufficient to involve them, and to reward them with a positive experience. For children and other sectors of society, it was felt important to introduce them to broader aspects of tree ecology, not least basic tree species identification, and elements of tree characterisation and measurement too. Previous OPAL surveys had succeeded when participants were asked to engage with the subject of the survey in appropriate ways for them.

A survey made of three separate but related components emerged from these discussions. The first component (Activity 1, “Get to know your tree”) was based around characterising the immediate environment around the tree chosen for the survey and asking the participant(s) to identify the tree, measure its girth and height and make observations on its general health or condition. A common method for assessing tree height based on simple trigonometry was devised. Instructions for assessing tree health or condition were developed from previous forestry protocols [6]. Survey questions about the tree’s environment served two purposes. Firstly, such information was considered useful in the subsequent analysis of tree P&D information generated by the survey. Secondly, it would help the survey planners in understanding where and in what parts of the country (e.g., town or countryside) participants felt more motivated to observe and record trees. Asking participants to measure the tree size was considered important scientifically so that other observations could be placed in context of the general size and age of the tree. It would also encourage engagement by offering a new skill to participants who had no previous experience in this aspect of tree survey. Information on previous experience of working with trees was also sought. Specific details of the protocol followed by citizens and the data they recorded are presented in Table 1.

The middle component of the survey (Activity 2, “Pests and diseases”) chose to focus on only three well known and loved tree species, for which it was highly likely that observations of P&D would be made. The P&Ds (Table 2) selected for surveillance were mainly well established on the chosen tree hosts, but observations on their latest distribution, impact and geographical extent would be scientifically valuable. Specific details of the survey protocol and the data recorded are presented in Table 3.

The final component (“The Most Unwanted”) focussed on P&Ds of concern to amenity and forest trees because of their damaging reputation from mainland Europe and beyond (Table 4). The purpose of the activity was to provide “early warning” information to officials. For the most part, these were non-indigenous species, although one, Chalara ash dieback (*Hymenoscyphus fraxineus*, formerly *H. pseudoalbidus*), emerged as a serious threat to ash trees in parts of Britain during survey development. A list of six P&Ds—“The Six Most Unwanted”—focussed on insect pests because these would be easier to spot. Specific details of the survey protocol and the data recorded are presented in Table 5.

The survey was supported by a suite of resources, including a field guide, a field notebook, a tree identification guide and a poster (Supplementary Figure S2), which was also available as an application for mobile devices. These resources were available both as a laminated “hard copy” survey pack, along with a tape measure and a pencil, and were downloadable from the OPAL website. In total, 50,000 survey packs plus 2000 Welsh language versions were distributed during the survey.

OPAL’s normal method of public engagement involved two approaches. Survey participants could either download a survey pack from the OPAL website and undertake the surveys completely independently, or more often, one of OPAL’s “community scientists” provided training and then organised and oversaw completion of a survey. Community scientists were staff with skills in engaging the public with science and nature, who worked in one of OPAL’s network of contracted partner universities and conservation organisations [16].

Prior to launch of the survey in May 2013, experts from Forest Research, Fera and OPAL delivered a series of 10 “train-the-trainer” sessions across England and with one session in Scotland and one session in Wales. “Trainers” were themselves trained to cascade training further within their own territories, organisations or volunteer groups. Around 300 “trainers” from a wide range of organisations (government agencies, local government, schools, national and local conservation and volunteer groups) were trained at these sessions. Included in the numbers trained were OPAL’s existing network of community scientists across England. In advance of a UK-wide launch in May 2015, further training was also provided to a new cadre of OPAL community scientists which were based in organisations in Scotland, Wales and Northern Ireland. Finally, given the specialist nature of trees and tree health, a “buddy scheme” was used in which people involved professionally with trees were available to assist the public in conducting the survey.

Participants were required to report results of their surveys either directly to the OPAL website or by posting completed surveys to OPAL. Except for suspected sightings of the “The Most Unwanted,” which were verified by FR scientists, no other records were verified by experts. However, the on-line survey form did include two “test” questions—a photograph of an ash twig and a horse-chestnut compound leaf—to provide an indication of the level of the participant’s tree knowledge. Participants entering their results on-line were also invited to complete a short questionnaire to explore effects of the survey on their learning, attitudes and behaviours (Table 6).

The survey remained open for data entry for the period 2013–2019, after which it was closed for further data entry. However, the survey resources have remained accessible to anyone wishing to undertake the survey for their own interest [18].

## 3. Results

### 3.1. Public Engagement and Awareness Raising

**Figure 1:**
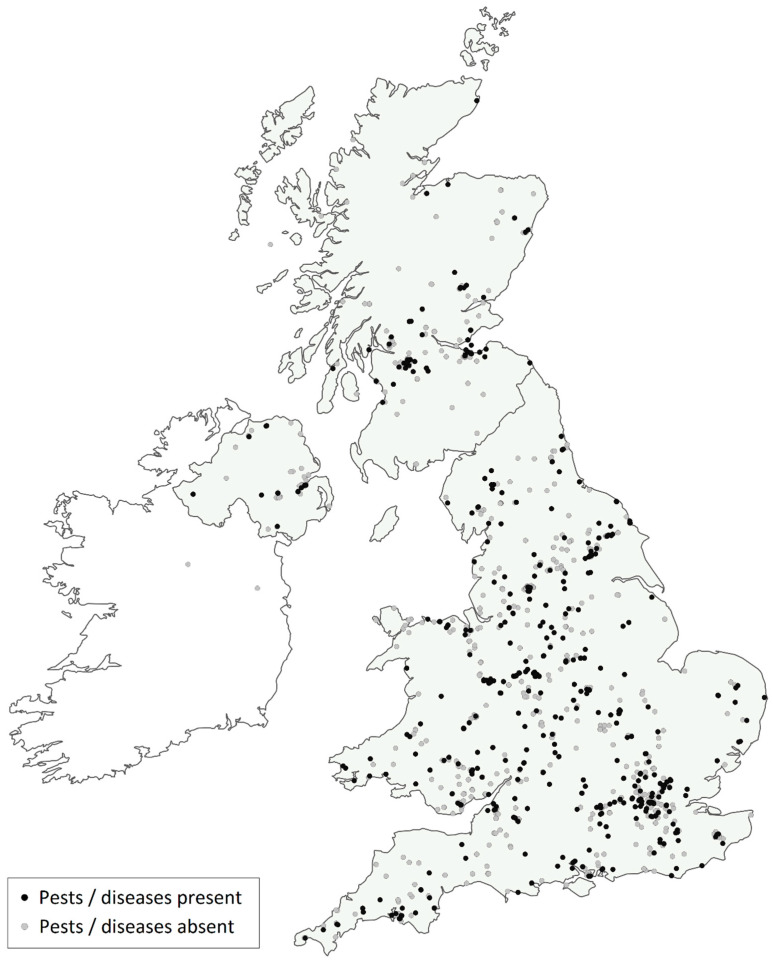
Map of completed surveys and “positive” P&D records.

An estimated 39,000 participants completed and submitted 2807 surveys involving 4505 trees. A majority of surveys (1148; 41%) were completed in the first year (2013), with much lower numbers of between 163 and 348 being completed in subsequent years up to 2019. Surveys were completed across the whole of the UK: England (63%), Scotland (25%), Wales (6%) and Northern Ireland (5%) (Figure 1). There was roughly a 60:40 split between surveys being completed by educational institutions (primary schools 25%, secondary schools 31% and universities/colleges 3%) and by the general public (friends and family 21%, adult volunteer groups 16% and youth groups 0.5%). The vast majority of participants obtained their survey pack at a school lesson (48%) or an event (19%) run by an OPAL community scientist. In contrast, only 18% of participants downloaded the survey pack from the OPAL website. Only 2% of surveys were completed as part of “buddy” scheme. Not unexpectedly, given the popularity of the survey with schools, 83% of participants reported having no previous experience of working with trees. A significant proportion (13%) had worked with trees through a volunteer group or society and 4% of participants worked with trees professionally. In the two tests of expertise, 82% of participants correctly identified an ash twig and 92% correctly identified a horse-chestnut leaf. Highly positive responses were indicated to the additional questions on learning (“Learnt something new,” 86%, N = 2132; “Developed new skills” 84%, N = 2131); willingness to continue involvement (“Will sign-up to a new survey” 79%, N = 989; “Recommend OPAL to friends and family,” 78%, N = 1095); and attitudes/behaviours towards the environment (“Changed attitude to the environment” 64%, N = 2090; and “Changed behaviour to the environment,” 60%, N = 991).

### 3.2. Trees and General Tree Health

#### 3.2.1. Location of Survey Trees

Nearly 60% of trees were identified as from streets, schools, parks or gardens, suggesting that volunteers chose to survey trees relatively close to their residences or places of study. Just over 30% of trees were identified as being from hedgerows and woodland, and these, too, could reflect an urban as much as a rural location. Trees in open fields constituted only 5% of the total number of trees surveyed.

#### 3.2.2. Tree Species

**Figure 2:**
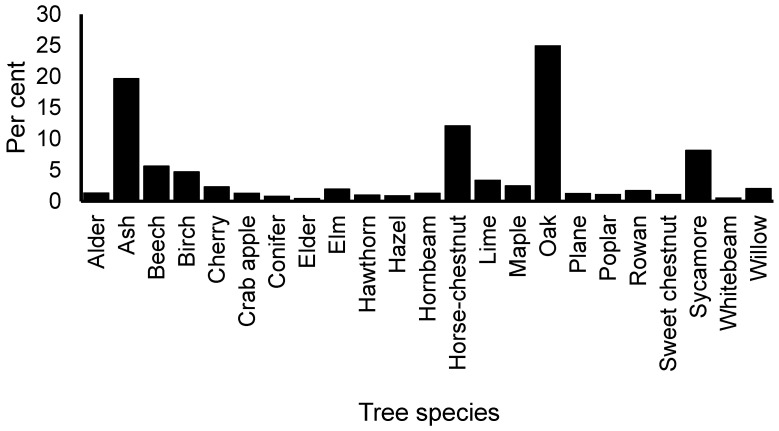
Range and frequency of tree species recorded in the UK OPAL tree health survey.

Figure 2 shows the tree species selected for assessment by the OPAL volunteers. In the context of urban tree populations, the range and frequency of species seem reasonably representative compared to those identified previously in the Trees in Towns II survey (TiT2) [3]. The relatively large reporting of oak, ash and horse-chestnut is probably partly an artefact of the survey protocol, which asked volunteers to focus on those three species when observing specific pests and diseases. Conifer species were seemingly under-reported compared to species as identified for England via TiT2, and this may reflect a perception of unattractiveness or unfamiliarity amongst some surveyors, rather than low numbers of conifers per se. The lack of Leyland cypress records in the OPAL survey (compared to TiT2) may be explained by its preponderance as a hedgerow former rather than its occurrence as an individual tree—the latter category probably lent itself to survey rather than hedgerow trees where it was difficult to access and take the measurements and assessments required by the survey protocol. This might also explain the poor recording of hawthorn. Cherry is another genus under-represented in the OPAL survey compared to TiT2, though it is unclear why this is so, and it might indicate a reduction in the population from 2008. In general, however, it is difficult to make temporal comparisons between the two surveys because of differences in protocol between them.

Compared to earlier FR volunteer surveys [8,9], there was a broad similarity in species occurrence, though lime is seemingly under-reported in OPAL compared to FR. Again, this was likely to be due to differences in protocol. The results suggest that without a detailed protocol on species selection, OPAL volunteers chose to cover many major tree species occurring in urban centres, but with some serious omissions. However, it is likely that for many of the minor species there is likely to be insufficient data to generate useful spatial pictures of the distribution of tree health and condition.

#### 3.2.3. Tree Canopy Density

**Figure 3:**
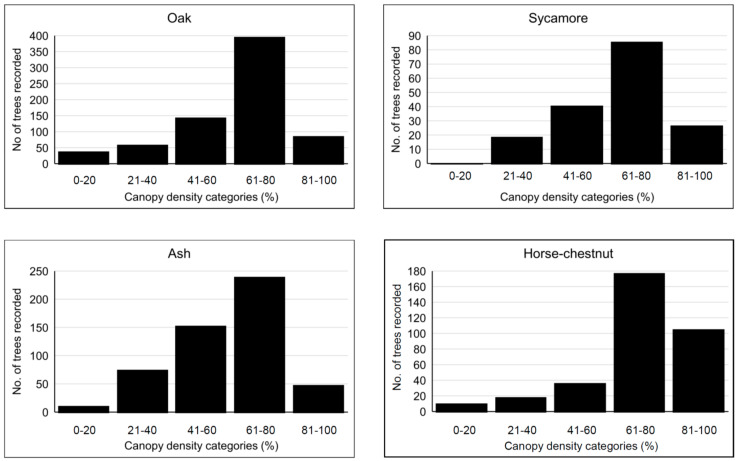
Frequency of occurrence of canopy density in five classes for the four most commonly recorded tree species in England only.

The four main tree species recorded by OPAL surveyors (oak, ash, horse-chestnut, sycamore) demonstrate very similar average tree canopy density profiles (see Figure 3), and all four have the majority of trees with 60–80% canopy density. The canopy density profile for oak can be compared with the distribution maps of oak canopy density in forests produced by Forest Research from 2000–2006 [19]. The results are broadly comparable in spite of different surveying protocols and the fact that over 50% of the oak OPAL records are from towns and cities. Compared with oak, ash exhibited comparatively poor canopy density, with many more trees showing low density. Horse-chestnut exhibited the best density profile overall, reflecting its greater leafiness, and its tolerance to the urban environment.

#### 3.2.4. Tree Leaf Browning

Despite the outbreak of Chalara ash dieback in 2012, Figure 4 suggests that ash was relatively unaffected by leaf browning. This can probably be explained by the fact that two thirds of the records for ash were made in the first two years of the survey (2013–2014) when the prevalence of ash dieback was low (<20% of that known in 2020 [20]. Oak, too, was comparatively unaffected by leaf browning, especially compared to the two, non-native, species of horse-chestnut and sycamore. Horse-chestnut was particularly affected, probably reflecting the widespread incidence of main P&Ds it suffers from (see Supplementary Table S3). However, sycamore also appeared significantly affected too. This may be due to occurrence of *Rhytisma acerinum* (Tar spot) and/or the *Cristulariella depraedans* fungus. Gosling et al. [21] have recently described the widespread distribution of the former on sycamore across England as a result of another OPAL survey focussing on air pollution.

#### 3.2.5. Wildlife

**Figure 5:**
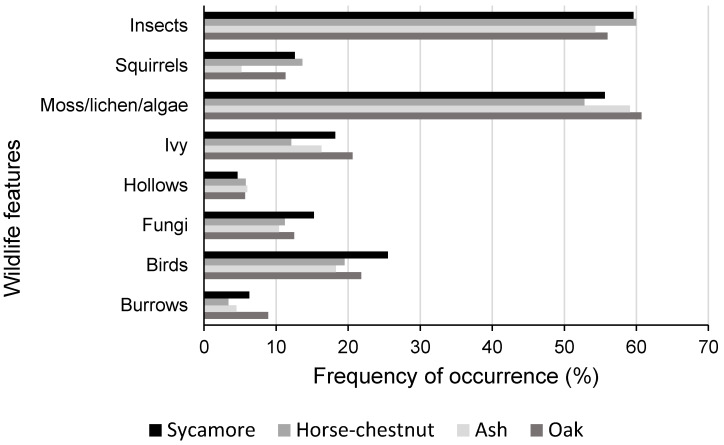
Frequency of occurrence of wildlife features recorded in the UK OPAL tree health survey.

The survey was designed so that surveyors could make several, easy-to-make, observations about aspects of wildlife associated with the trees under inspection, and 79% of records contained information on one or more aspects (Figure 5). The significant level of recording and collective identity of wildlife features suggests that trees, including urban trees, make a notable contribution to provision of habitat and biodiversity. There were few obvious differences between the four major species, such that it was not possible to discriminate between native and non-native species. Squirrel presence (presumably dominantly grey squirrel (*Sciurus carolinensis*)) appears to be very similar across all four species, bearing out our understanding of the tree species preference for these species [22].

### 3.3. Surveillance for Tree Pests and Diseases

#### 3.3.1. Established Pests and Diseases

**Figure 6:**
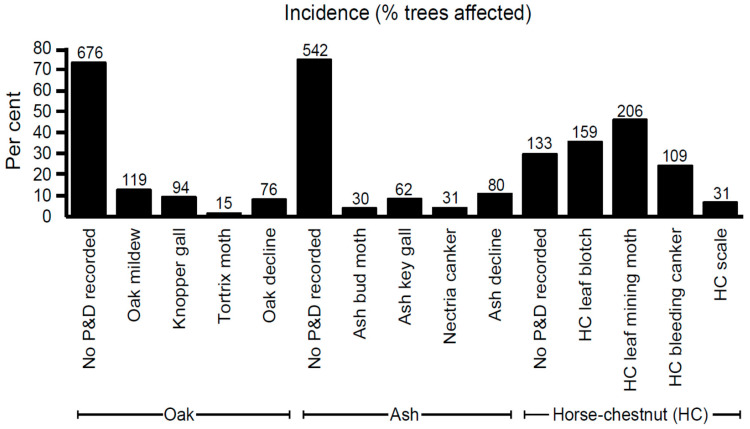
Incidence of selected pests and diseases recorded on oak, ash and horse-chestnut. Numbers above each column are actual number of records for the specific attribute.

The aim of this activity was to provide information on the latest distribution in the country of selected P&Ds of oak, ash and horse-chestnut. Results are presented in Figure 6. However, they need to be treated with some caution—although ease of identification by the public was a key criterion in selection of the P&Ds, none of the reports were verified by experts. In addition, the numbers of observations only allow for overall incidences to be presented. For all P&Ds, incidence will vary not just by year but also throughout a year and from one region of the country to another.

The survey provided observations of P&Ds on a total of 923 oak trees, 728 ash trees and 447 horse-chestnut trees. No P&Ds were recorded on 73% of oak trees and 75% of ash trees, but only 30% of horse-chestnut trees. On oak, mildew, knopper gall and decline were observed regularly (13%, 9% and 8%, respectively). In contrast, tortrix moth was found on only 2% of oak trees. On ash, decline and key gall were observed regularly (11% and 9%, respectively), whereas *Nectria* canker and ash bud moth were observed less often (4% and 4%, respectively). In contrast, incidence of P&Ds on horse-chestnut was much higher: leaf mining moth (46%), leaf blotch (36%) and bleeding canker (24%). Scale (7%) was the least observed pest.

Noting the aforementioned limitations of the OPAL data and the general scarcity of published data on the incidence of tree P&Ds, it was only possible to make a few comparisons between OPAL records and other datasets. In 2007, Forestry Commission conducted a survey of over 2500 horse-chestnut trees across Great Britain and found overall 49% trees were affected by horse-chestnut bleeding canker, 54% in urban environments and 44% in rural environments [23]. One long-term study in South East England showed the incidence of bleeding canker on horse-chestnut to increase from 33% in 2003 to 49% in 2012 [24]. Compared to these studies, the 24% incidence of horse-chestnut bleeding canker in the OPAL survey was low. However, there is anecdotal information that the incidence of horse-chestnut bleeding canker has reduced in recent years, as diseased trees have died or been removed.

The most northerly record of horse-chestnut leaf mining moth in the OPAL data set was a report from Pencaitland, East Lothian (Ordnance Survey (OS) NT46) in November 2014. “Conker Tree Science,” a citizen science project that also collects data on the distribution of horse-chestnut leaf-mining moth, reports the most northerly verified record to be near Aberfeldy, Perthshire (OS NN75) in January 2014. Other unverified records in Scotland were near Dyce, Aberdeenshire (OS NJ81) in August 2014, Aberdeen, Aberdeenshire (OS NJ90) in August 2014 and near Cumnock, Ayrshire (OS NS52) in September 2014 [25]. Horse-chestnut leaf mining moth was first recorded in the UK in Wimbledon in 2002 [26,27] and so a spread of 538 km in 12 years to Pencaitland would equate to 45 km per year, which is within published ranges of spread of between 20–36 km per year local spread and 143 km per year long distance spread [28] and 40–70 km per year [29,30].

In 1987, FC conducted a national survey for dieback on ash and oak [31]. These diseases correspond to ash decline and oak decline in the OPAL survey. The FC survey recorded an incidence of 19% for ash dieback (on 4454 ash trees surveyed) and an incidence of 18% for oak decline (on 1022 oak trees surveyed). OPAL recorded broadly comparable incidences of 11% for ash decline (on 728 ash trees surveyed) and 8% for oak decline (on 923 oak trees surveyed).

#### 3.3.2. Quarantine Pests and Diseases—“The Most Unwanted”

On the OPAL database, there were 28 suspect reports of “The Most Unwanted”: citrus longhorn beetle (three records), Asian longhorn beetle (one record), oak processionary moth (five records), pine processionary moth (one record), emerald ash borer (three records), and Chalara ash dieback (15 records). Forest Research experts checked reports sent to them in 2013. Except for nine Chalara reports involving 13 trees, all other records were deemed not to be credible mostly because of obvious errors by the participant, including a record on a non-susceptible host tree and multiple pest records on the same tree. Some Chalara findings were in areas where the disease had already been found, for example Kinghorn, Fife (2013), and so did not require further investigation. However, suspect reports from Hildenborough, Kent (OS TQ54; 2014), Sevenoaks, Kent (OS TQ55; 2014), Plymouth, Devon (OS SX55; 2016), Brougham, Cumbria (OS NY52; 2015), Bolton, Greater Manchester (OS SD70; 2014) and Newport, South Wales (OS ST28) could have represented the first time that Chalara was observed in these locations had they been confirmed by a physical sample. Consequently, the Ordnance Survey 10 km grid squares on the official Chalara outbreak map [32] did not turn positive until one or two years later.

People engaged on the OPAL project were also involved in two confirmed findings of Chalara. One was found by an OPAL “trainer” near Scrayingham, North Yorkshire in April 2015 (OS SE76) and the other by a community scientist in Brecon, Powys in 2015 (OS SO02). Samples from both findings were submitted to Fera, who confirmed the presence of the pathogen *Hymenoscyphus fraxineus*. The Brecon finding represented the first time Chalara had been confirmed in a 10 km grid square (SO02) on the official Chalara outbreak map. However, at the time of finding in 2015, the official identification was not communicated from Fera to FC. This meant that the first finding of Chalara in Brecon appeared on the official outbreak map to be 2017. The FC have subsequently updated the map to reflect this OPAL finding in 2015. Specific records of suspect sightings of “The Most Unwanted” do not exist on the OPAL database after June 2015.After this time, participants were only required to tick “yes/no” if they had seen a suspect “Most Unwanted” (21 “yes” records were made) and report sightings on Forest Research’s “TreeAlert” portal [33], which does not record the affiliation of a person making a record.

**Figure 7:**
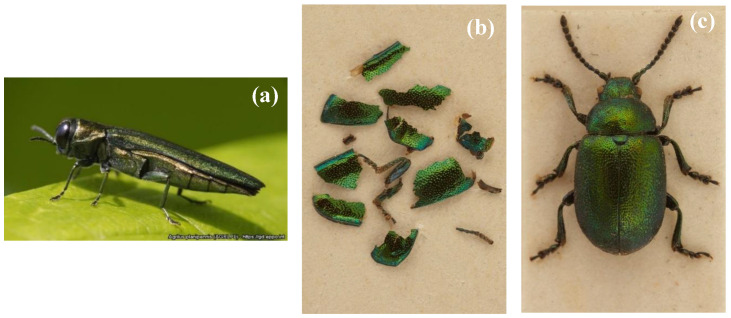
Fragments suspected to be emerald ash borer but actually green dock beetle. (**a**) Emerald ash borer (Courtesy: Eduard Jendek); (**b**) suspect emerald ash borer (UK Crown Copyright, Courtesy of Fera); (**c**) green dock beetle (UK Crown Copyright, Courtesy of Fera).

One other suspect sighting in 2013 is also worthy of mention. A survey participant captured a green beetle on an ash tree, which she suspected to be an emerald ash borer. She sent the beetle by post to OPAL, who duly sent the specimen, by now only fragments, to Fera. Expert diagnosticians at Fera confirmed the beetle to be a green dock beetle (*Gastrophysa viridula*) and not emerald ash borer (Figure 7).

## 4. Discussion

The OPAL Tree Health survey project sought to determine the effectiveness of a citizen science approach in contributing to government tree health public engagement and surveillance needs. Whereas the approach was considered largely to be a success in terms of public engagement, it was deemed to be only of limited success in generating useful data on specific tree pests and diseases. We discuss these findings below.

### 4.1. Public Engagement

Several elements were considered to have contributed to the engagement success, including:

#### 4.1.1. Inspiring a New Generation of Environmentalists

The project engaged a large number of people (estimated to be over 39,000), especially the younger generation, with the topic of trees, tree health and specific pests and diseases, giving many participants their first experience of the subject. Furthermore, responses to evaluation questions (Section 3.1) confirmed that participation in a scientific survey helped people not only to learn something new and develop new skills but it also positively influenced their attitudes and behaviours towards the environment.

#### 4.1.2. Building Partnerships

Through its survey Working Group and the survey Advisory Board, the project brought together a broad consortium of universities, governments, government agencies, non-government organisations, forest managers and conservation groups. This collaborative approach contributed to the quality of the survey methodology, supporting materials and the uptake of the survey. In addition, many of the people and institutions also remained involved in tree health, helping to form interdisciplinary groups such as the Tree Health Citizen Science Network and Defra’s Tree Health Policy Group. Indeed, this is a tangible outcome of the “Action Research” approach [34] adopted in the OPAL project.

#### 4.1.3. Innovation and Inclusion

OPAL’s innovative approach was to make activities and resources engaging and accessible so that the complex science of tree health was understandable and the experience of participation was rewarding to all, regardless of age, background or ability. Essential to this process was the provision of text that was scientifically accurate, concise and easy-to-understand and by producing supporting materials that were high-quality and visually appealing (e.g., field guide, field notebook, tree identification guide and poster). Inclusion was facilitated by making the materials available both in hard-copy and on-line, and also by producing Welsh language versions for use in Wales (Supplementary Figure S2). In an annual award scheme run by the UK Government Department with responsibility for tree health (Department for Environment, Food and Rural Affairs—Defra), the OPAL Tree Health Survey won the award for “Civil Service Reform” in 2013 in recognition of its innovative approach to engage the public actively in support of wider government policy, in this case safeguarding tree health.

#### 4.1.4. Support

Most of the engagement was facilitated by a national network of local group leaders who provided face-to-face training, delivered lessons to school children and then often oversaw completion of surveys in the field and subsequent data entry back in the classroom. This local contact was largely achieved through OPAL’s contracted network of community scientists but also involved public engagement staff in official agencies, such as FC and Fera. The results of the survey and the feedback submitted (Section 3.1) demonstrate the success of this approach.

### 4.2. Surveillance for Tree Pests and Diseases

The project successfully catalysed the completion and submission of over 2800 surveys of more than 4500 trees from across the whole of the UK, possibly at a scale beyond the scope of the limited number of officials, such as plant health and forestry inspectors. Over two thousand oak, ash and horse-chestnut trees were surveyed specifically for pests and diseases and over one thousand records of pests and disease were made on these trees. The project also successfully generated “negative records” of the lack of specific P&Ds, which might not normally be of interest to the public but could be important to scientists. The recorded incidences of the various P&Ds were compatible with other studies where comparison was possible, for example horse-chestnut bleeding canker [35] and horse-chestnut leaf mining moth [36]. Some credible reports of suspect quarantine pests and diseases, mostly of Chalara ash dieback, were also submitted, and one OPAL Chalara report (Brecon, Powys) changed the official outbreak map. In addition, one of the false positives, the suspect emerald ash borer that turned out to be green dock beetle, provided proof of concept that some members of the public could be engaged in official surveillance. Taken together, these results demonstrate that citizen scientist projects like OPAL can generate scientifically important information.

However, several challenges were experienced, which meant that, overall, the survey was considered only to have been of limited success in generating useful data on specific tree pests and diseases of value to those charged with strategic surveillance. These challenges are discussed in more detail below with the aim to inform and improve future tree health citizen science projects.

#### 4.2.1. Data Entry Errors and Lack of Verification

Notwithstanding the care given to select pests and diseases that were considered to be relatively easy for the public to identify, and all the support provided to participants (face-to-face training, supervision during surveying, identification guides, etc.), numerous data errors were encountered. Some were simple recording errors, such as impossible values for tree height or girth and others were not credible, e.g., a pest record on a non-host tree. Such problems could be avoided in future through tighter constraints on data entry (e.g., dropdown lists with a range of numeric values of specific units within normal expectations), by functionality to flag a warning if a pest was recorded on a non-host or by inclusion of a requirement for a photograph and triaging or verification by an expert. These refinements will require significant input, but they are considered vital to reduce the generation of spurious or inaccurate data, which can reduce the overall trust that scientists and officials place in the project. Aceves-Bueno et al. [37] further reflected on how to improve the accuracy of data derived by CS.

#### 4.2.2. Data Processes

Although OPAL’s previous surveys used tried and tested systems of on-line data entry, storage, visualisation and extraction, the demands associated with quarantine pests necessitated a manual process to identify and communicate records of “Most Unwanted” pests. Consequently, delays and omissions were encountered between OPAL and official agencies and between official agencies. One consequence of the latter resulted in inaccuracies in the official Chalara outbreak map operated by FC. The risk was mitigated by requiring OPAL participants also to enter their observations into the official national “TreeAlert” portal [33], but there was no provision in it for identifying the data provider as an OPAL surveyor. Our experience in the OPAL project leads us to strongly suggest that any future surveys of this type must consider how data generated by volunteer citizen scientists will be evaluated for quality in a cost-effective manner, and how they can be made available in as near real-time as possible to scientists and officials responsible for tree health surveillance.

#### 4.2.3. Involvement of Experts and Officials

The involvement of experts and officials after the launch of the survey was less than necessary to ensure the survey yielded results of benefit to tree health surveillance. All suspect reports of “The Most Unwanted” were investigated, at least up to August 2013 and results of the first two years’ of the survey were summarised and published on the OPAL website. However, there was no expert monitoring of data of the established pests and disease on oak, ash and horse-chestnut. Several factors contributed to this lack of involvement, including a lack of capacity in official agencies, a lack of integration of the OPAL tasks into existing surveillance programmes and a large number of changes in personnel of officials and scientists, all of which was exacerbated by the heavy demands of the extensive programme of official surveillance and action against Chalara ash dieback. Although the OPAL tree health survey project originated out of policy directives to involve the general public more in such matters, our experience in devising and leading the project demonstrated that it is vital to gain support of the scientists and officials responsible for tree surveillance. Lakeman-Fraser et al. [38] discuss this in greater detail, pointing out that “buy-in” can be challenging in “scientific disciplines where citizen science is new, novel or perceived as threatening.”

Significantly, lessons learnt on the OPAL project were put into immediate effect on the Observatree Project [15] as many of the same people were involved. The latter project benefited from engagement of volunteers with existing knowledge of trees and tree pests and diseases, close and continued involvement of expert government scientists to triage, verify and act on reports, and better integration of sighting reports into official databases.

### 4.3. Can the Public Play a Useful Part in the Surveillance of Tree Health in the Future?

The successes and failures of the OPAL Tree Health Survey, along with the authors’ intimate experience of the project, provide the basis for proposing a blueprint for future involvement of citizens to support the needs of official surveillance for tree pests and diseases. Firstly, OPAL’s mixed success at achieving both public engagement and surveillance objectives demonstrates that scientists or policymakers considering a citizen science approach should be absolutely clear about the overall aim of the proposed activity: is the aim public information, public engagement or surveillance and science? Knowing the aim will then inform the decision on which approach to take and the depth of citizen involvement in the activity.

If the aim is “Public information” to raise public awareness of tree pests and diseases, a citizen science approach is not specifically required and instead these aims are probably better achieved through publicity campaigns using social media, television, radio, newspapers and journals; posters/leaflets in airports; films; videos; exhibits; show gardens at flower shows; arts and crafts, video games; etc. In contrast, if the aim is “public engagement” to develop the public’s knowledge and skills of trees in general and tree health more specifically, then citizens could be involved more actively. Engagement might involve practical field activities such as tree planting, but also citizen science activities that encourage citizens to learn about and identify tree species. Further involvement including making basic measurements and assessments (e.g., tree height, girth, crown density and presence of wildlife) can help consolidate interest and understanding. The OPAL Tree Health Survey (Activity 1) and Treezilla [39] are examples of such projects. Properly supported, these activities can also generate data to support surveillance and science.

Ultimately, if the aim is “Surveillance and science,” then citizen science could be a very suitable approach to generate data on the presence and incidence of tree pest and disease and provide early detection of new pests and diseases coming into the country/region. Should scientists/policymakers decide to involve citizens in support of surveillance for tree pests and diseases, the following requirements based on lessons learnt during the OPAL project are advised.

Firstly, people with existing knowledge of trees and tree pests and diseases are likely to generate more reliable data, requiring less verification and less resource to train and support. Currently, the Observatree Project, which has trained a national cadre of around 452 expert volunteers, is an example of where this approach has proved to be highly effective [40] It appears to be very similar in approach to that adopted in France [41]. Should the need exist to expand the surveillance network, it is suggested that this could be best achieved by involving professional people, such as arboriculturists and foresters, as they are already working with trees and many have specific knowledge of tree P&Ds. We recommend that those charged with further development of tree health surveillance systems undertake formal liaison with the professional bodies that represent these sectors in order to explore practical ways to implement this suggestion.

However, at critical times when additional surveillance capacity is required, e.g., to detect a new pest or pathogen incursion, the experience of the OPAL Tree Health Survey and other projects such as “Conker Tree Science” [42] is that non-expert citizens can contribute effectively if suitably instructed and guided. Such an approach is likely to be most effective for time-limited surveillance against a specific pest or disease when observations can be verified easily by provision of a photograph. Indeed, the authors see huge untapped potential in engaging with the biological recording community more broadly as they not only have similar interests in biodiversity but also their staff and volunteers already possess skills of observation, species identification and recording. Reciprocal sharing of such a resource is proposed as one way the UK Government might meet its target to train at least two per cent of the UK population (1.3 million) as biosecurity volunteers [43].

Secondly, a single, well publicized, user-friendly and easily accessible portal should be used to report pests and diseases under surveillance. For example, “TreeAlert” [33] is the portal currently supported by official forest agencies for use in Great Britain, whilst TreeCheck [44] is available in Northern Ireland. However, endorsement of “TreeAlert” by plant health authorities is unclear from the TreeAlert website (unlike for “TreeCheck”) and publicity of the portals by professional institutions is patchy—the keyword “TreeAlert” cannot be found by searching the two principal British forestry professional institution websites, though the main UK arboricultural organization offers several links to it. Consequently, we consider that this resource is underused compared to similar ones offered in Scandinavian countries, for example [45,46]. Further development of a single portal must include a component for ongoing communication and publicity. It should also include provision of access using smartphones and tablets. However, any expansion of its use to other professionals and professional organisations will require enhancements, for example to permit users to see their own information.

Thirdly, management of data submitted by citizen scientists must ensure real-time continuity with scientists or other officials charged with peer-reviewing them. The data management system should include functionality to provide surveyors with feedback that their contributions have been received and logged on the system. For example, the Norwegian and Swedish versions allow public access to all approved tree health reports, including the name of the surveyor. However, it is evident that the tree health database should sit inside government, or government agency rather than in any Citizen Science project or organisation.

Finally, to provide the most effective support to official surveillance, the programme of citizen activity needs to be “mainstreamed” into the programme of official surveillance, and receive adequate financial and technical support, including training and coordination. Training represents a significant investment, and therefore any citizen programme will require core rather than project-based funding to maximise longevity and give confidence, and status, to citizens who “sign up,” similar to that in France [41].

## 5. Conclusions

By engaging an estimated 39,000 people to complete and submit over 2800 surveys of more than 4500 trees in the UK, the OPAL experiment has demonstrated that the public can play a useful part in surveillance of tree health. Nevertheless, we consider that the approach was a greater success in terms of public engagement than surveillance. Whereas the OPAL survey successfully gave many participants their first experiences of trees, developed both capacity and capability and even fostered environmental stewardship, the data generated were of limited scientific value. Involving citizens with some existing expert knowledge is suggested as the most effective way to expand the surveillance network to generate more reliable data, thereby requiring less verification and less resources to train and support. However, less expert citizens, with suitable guidance and support, can contribute effectively at critical times when additional surveillance capacity is needed. Collaboration across the spectrum of citizens who already observe, identify and record other species is another approach which should be examined. To be most effective, citizen activity needs to be “mainstreamed” into the official surveillance programme, using a single reporting portal which feeds into integrated database(s), and needs to receive adequate financial, technical and political support.

## Figures and Tables

**Figure 4:**
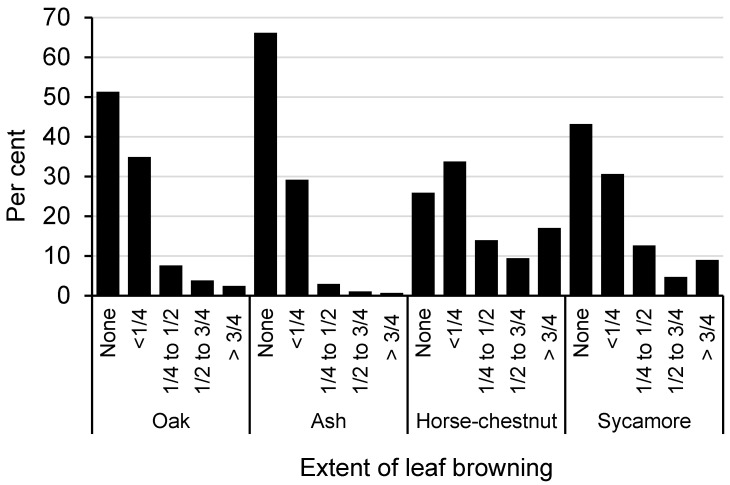
Frequency of occurrence of leaf browning for the four most commonly recorded tree species in England only.

**Table 1:** Summarised recorded variables for Activity 1 “Get to know your tree.”

Question Category	Answer Variables
Have you carried out a field survey before this one?	Yes, No
1. Date of survey	dd/mm/yyyy format
2. Who did you do the tree health survey with?	Primary school, Secondary school, Youth group, Adult Volunteer group, Family or friends, College/university, Other (free text), buddy
3. Are you involved in working with trees or forestry?	No, Yes, as part of a volunteer group or society, Yes, I work in the industry
4. Record the location of your tree	Latitude/Longitude (automatically calculated) from address, postcode or click location on map
5. Which of these best describes your survey area?	Street, Garden, School, Park, Open field, Hedge, Edge of woodland, Inside woodland, Other (free text)
6. What is the ground cover around the base of the tree?	Grass or other plants, Bare soil, Hard surface, Fallen leaves
7. If there are fallen leaves beneath the tree, how many are there?	A few, A lot, Ground completely covered
8. Record the name of the tree (tree species)	Drop down list provided, other (free text)
9. Measure the girth (circumference) of the trunk at 1.3 m (130 cm) above the ground	Numbers only (cm)
10. Measure the height of the tree	Numbers only (m)
11. Is the tree taller or shorter than most other trees nearby?	Shorter, Taller Same Hight, No trees nearby
12. Which of these best shows the shape of your tree?	Graphics given for: Spreading, Oval, Fan, Column, Cone
13. Estimate how much of the view through the crown is made up of leaves.	No leaves, 25%, 50%, 75%, All leaves
14. Can you see any dead wood (branches that have no leaves or twigs on them)?	Yes, No If Yes, Less than a quarter, Between one quarter and one half, Between one half and three quarters, More than three quarters
15. What types of leaf browning can you see on the tree?	Tick all that apply: Brown leaf edges, Brown spots, Leaves all brown, none
16. If there is leaf browning, how much can you see?	Less than a quarter, Between one quarter and one half, Between one half and three quarters, More than three quarters
17. What types of leaf yellowing can you see on the tree?	Tick all that apply: Yellow leaf edges, Yellow spots, Leaves all Yellow, none
18. If there is leaf yellowing, how much can you see?	Less than a quarter, Between one quarter and one half, Between one half and three quarters, More than three quarters
19. Can you see any of the following signs of insect damage on the leaves?	Check any that apply: Leaf holes, Leaf mining, Leaf galls, No damage
20. Record any animals, signs of animal activity, plants or fungi	Birds or birds’ nests, Burrows at base, Fungi, Hollow, Ivy, Mosses or lichens, Squirrels, Spiders or insects, Other
Medal Award	Based on a calculation of the previous entries, the tree was awarded a medal: Gold, Silver, Bronze, No medal

**Table 2:** Pests included in survey to provide information on their latest distribution.”

Common Name	Latin Name	Quarantine Status in 2013
Pests and diseases of oak (*Quercus* spp.)		
Knopper gall	*Andricus quercuscalicis*	Present; first found in 1950s
Tortrix moth	*Tortrix viridana*	Present; native
Oak mildew	*Erisiphe alphitoides*	Present; first found in 1908
Oak decline	Caused by a combination of pests and diseases; *Agrilus* oak beetles are attracted to the weakened trees	Present
Pests and diseases of ash (*Fraxinus excelsior*)		
Ash bud moth	*Prays fraxinella*	Present; native
Ash key gall	*Aceria fraxinivorus*	Present; native
Nectria canker	*Neonectria galligena*	Present; native
Ash decline	Caused by a combination of factors that affect roots and cause decline	Present
Pests and diseases of horse-chestnut (*Aesculus hippocastanum*)		
Horse-chestnut leaf miner	*Cameraria ohridella*	Present; first found in 2002
Horse-chestnut scale	*Pulvinaria regalis*	Present; first found in 1964
Horse-chestnut leaf blotch	*Guignardia aesculi*	Present; first found in 1935
Horse-chestnut bleeding canker	*Pseudomonas syringae* pathovar *aesculi*	Present; first found in early 2000s

**Table 3:** Summarised recorded variables for Activity 2, “Pests and diseases.”

Question Category	Answer Variables
Did you spot any Oak pests or diseases?	Yes, No
If yes, is this the tree surveyed in Activity 1	Yes, No
Which Oak pests or diseases did you spot? Check any that apply.	Oak mildew, Knopper gall, Tortrix moth, Oak decline
	Please upload a photo of the Oak disease(s) you have found
Did you spot any Ash pests or diseases?	Yes, No
If yes, is this the tree surveyed in Activity 1	Yes, No
Which Ash pests or diseases did you spot? Check any that apply.	Ash bud moth, Ash key gall, Nectria canker, Ash decline
	Please upload a photo of the Ash disease(s) you have found
Did you spot any Horse-chestnut pests or diseases?	Yes, No
If yes, is this the tree surveyed in Activity 1	Yes, No
Which Horse-chestnut pests or diseases did you spot? Check any that apply.	Horse-chestnut leaf blotch, Horse-chestnut leaf miner, Horse-chestnut bleeding canker, Horse-chestnut scale
	Please upload a photo of the Horse-chestnut disease(s) you have found

**Table 4:** Pests included in survey to provide “early warning” information (based on details presented in OPAL’s Tree Pest and Disease Identification Guide).

Common Name	Latin Name	Threat	Quarantine Status in UK (2013)
Asian longhorn beetle	*Anoplophora glabripennis*	Major threat to a wide range of broad-leaved trees, especially *Acer* spp. Most damage caused by larvae tunnelling through wood.	Not yet established; single outbreak found in 2012 (now eradicated) [17]
Citrus longhorn beetle	*Anoplophora chinensis*	Major threat to a wide range of broad-leaved trees, especially *Acer* spp. Most damage caused by larvae tunnelling through wood.	Not present; occasional interceptions on imported trees
Emerald ash borer	*Agrilus planipennis*	Major threat to ash (*Fraxinus* spp.). Most damage caused by larvae tunnelling through wood.	Not present
Oak processionary moth	*Thaumetopoea processionea*	Caterpillars cause severe defoliation of oak (*Quercus* spp.) and pose a human health risk from toxic irritating hairs.	Very limited distribution in the UK; first found in 2006
Pine processionary moth	*Thaumetopoea pityocampa*	Caterpillars cause severe defoliation of pine (*Pinus* spp.) and pose a human health risk from toxic irritating hairs.	Not present in the UK
Chalara ash dieback	*Hymenoscyphus fraxineus*	Major threat to ash (*Fraxinus* spp.). Fungus causes defoliation, dieback and death.	Limited distribution in the UK

**Table 5:** Summarised recorded variables for Activity 3, “Most Unwanted.”

Question Category	Answer Variables
Have you found any of our Most Unwanted? Check any that apply.	Yes, No Asian longhorn beetle, Citrus longhorn beetle, Emerald ash borer, Oak processionary moth, Pine processionary moth, and Chalara ash dieback
Did you survey a second tree at the same location on the same date?	Yes, No If yes, questions repeat
Did you survey a third tree at the same location on the same date?	Yes, No If yes, questions repeat

**Table 6:** Evaluation questions.

Question Category	Answer Variables
Where did you get your survey pack?	Downloaded from OPAL website, OPAL scientist/OPAL event, (NFWI Wales Tree Project), Other (free text)
Did you learn something new about your local environment?	Yes, No
Have you developed new skills?	Yes, No
Would you recommend OPAL to your family and friends?	Yes, No
Do you feel that taking part in this survey has changed the way you think about the environment?	Yes, No
Will you change your behaviour towards the environment?	Yes, No
Comments	Free text

## Supplementary Materials

The following are available online at https://www.mdpi.com/2075-4450/11/9/550/s1. Figure S1: First arrival of damaging tree pests and pathogens to the UK, 1970–2020 [47]. Figure S2: Examples of OPAL Tree Health Survey resources in English and Welsh. Table S1: OPAL Tree Health Survey Working Group partners and responsibilities. Table S2. OPAL Tree Health Survey Advisory Board partners and responsibilities.
