# Supplementary material for: How Effective Are Citizen Scientists at Contributing to Government Tree Health Public Engagement and Surveillance Needs—An Analysis of the UK Open Air Laboratories (OPAL) Survey Model
Source: Insects. 2020 Aug 19;11(9):550. doi: 10.3390/insects11090550 (PMC7563208; doi:10.3390/insects11090550)
Supplement: Supplementary file 1 [file insects-11-00550-s001.zip › insects-871409-supplementary.docx]

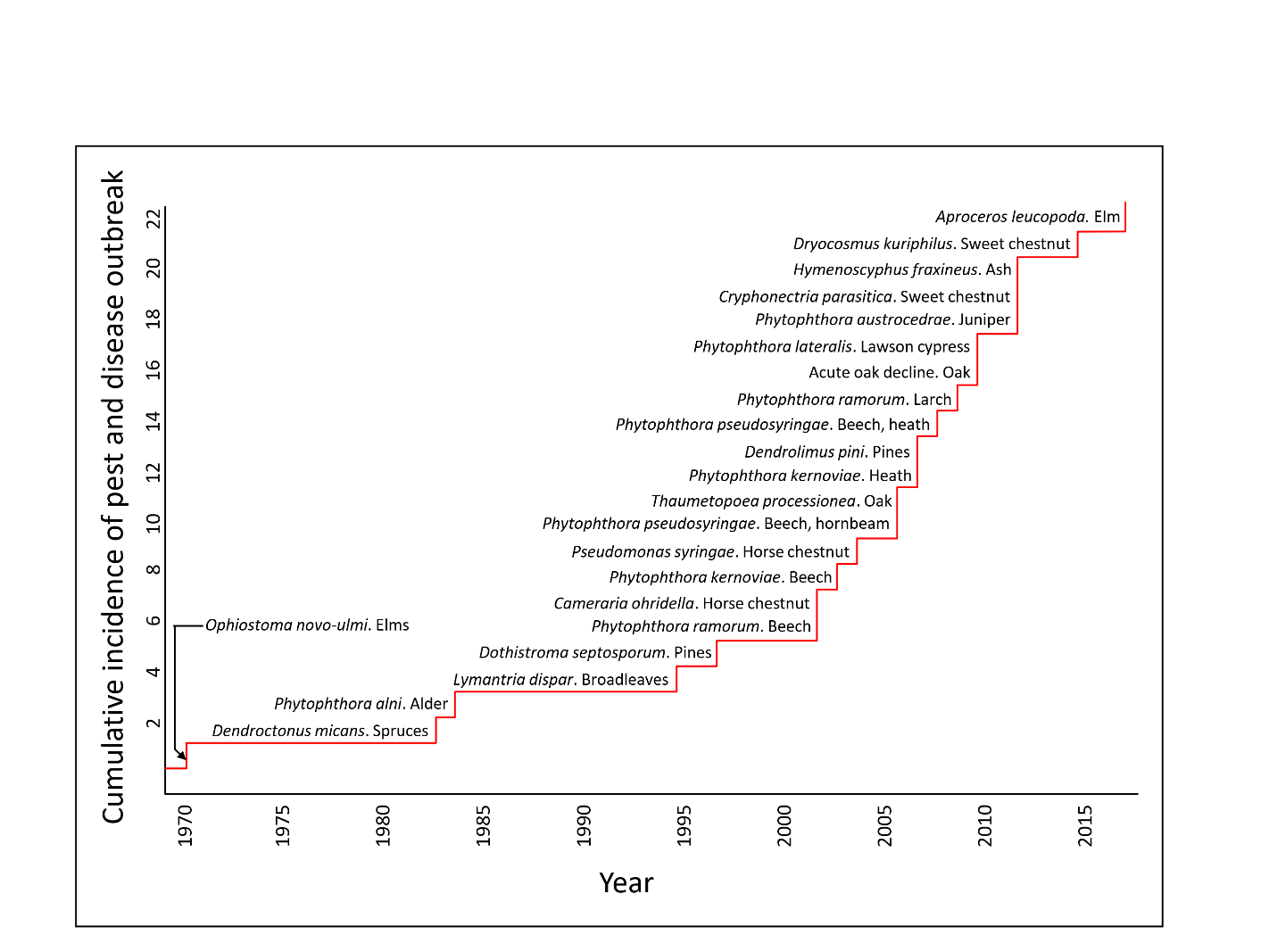


**Supplementary Figure S1.** First arrival of damaging tree pests and pathogens to the UK, 1970-2020 (Figure prepared by the authors and based on information from Forestry Commission [47]).

| 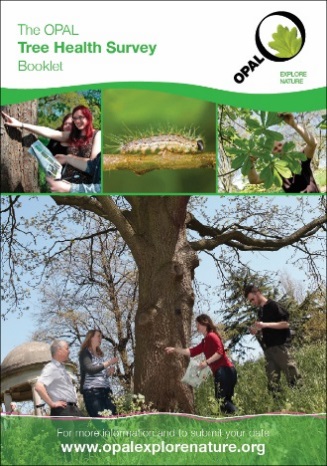 | 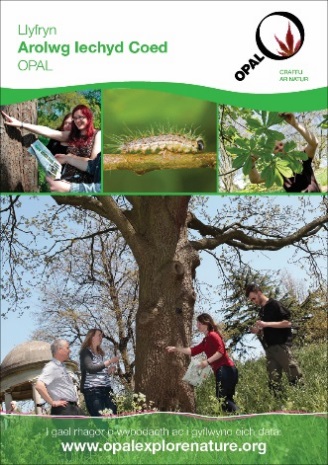 | 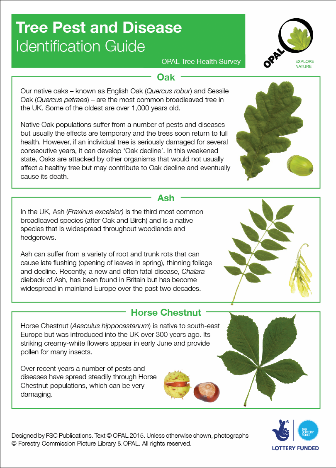 | 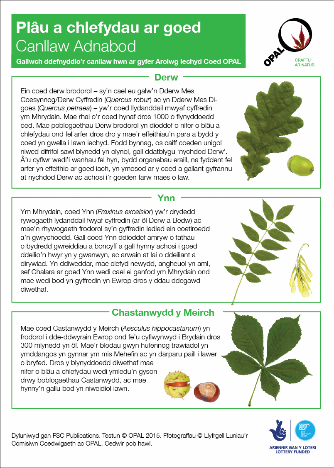 |
| --- | --- | --- | --- |

**Supplementary Figure S2.** Examples of OPAL Tree Health Survey resources in English and Welsh.

| Organisation | Organisation type | Role/responsibility in survey |
| --- | --- | --- |
| Food & Environment Research Agency | Government agency (research and regulation) | Governance (chair); technical expertise; training; press and media support |
| Forest Research | Government agency (research) | Technical expertise (lead); drafting survey materials and images; training; press and media support |
| OPAL Imperial College | University | Project management (lead) o-ordinate and oversee governance, development, printing and distribution of survey packs; website  data entry, storage and transfer to Forest Research; press and publicity; organise training; network of Community Scientists to deliver surveys across the country |
| Field Studies Council | Charity (environmental educational) | Production and printing of survey materials |
| University of York | University | Practical advice; testing; training; user |
| Plymouth University | University | Practical advice; testing; training; user |
| Natural History Museum | Non-Departmental Public Body | Website and database |

**Supplementary Table S1.** OPAL Tree Health Survey Working Group partners and responsibilities.

**Supplementary Table S2.** OPAL Tree Health Survey Advisory Board partners and responsibilities.

| Organisation | Organisation type | Role/responsibility in survey |
| --- | --- | --- |
| Food & Environment Research Agency | Government Agency | Governance (chair) |
| Forest Research | Government Agency | Technical lead |
| OPAL Imperial College, London | University | Project Management lead |
| Department for Food, Environment and Rural Affairs | Government Department | Policy oversight in England |
| Forestry Commission Scotland | Government Department | Policy oversight in Scotland |
| Forestry Commission England | Government Department | Policy oversight in England |
| Welsh Government | Government | Policy oversight in Wales |
| Woodland Trust | Charity (woodland conservation) | Practical advice/user |
| National Trust | Charity (Conservation) | Practical advice/user |
| Sylva Foundation | Charity (woodland conservation) | Practical advice/user |
| Tree Council | Charity (tree conservation) | Practical advice/user |
| Royal Parks | Government agency | Practical advice/user |
| Duchy of Cornwall | Crown | Practical advice/user |
| University College London | University | Observation and research of project |
